# Current status and influencing factors of telehealth readiness among nurses: A multicenter cross-sectional study in Ningxia, China

**DOI:** 10.1371/journal.pone.0343263

**Published:** 2026-02-19

**Authors:** Fang Yu, Jie Zhao, Xiaona Zhang, Tingting Li, Hongyan Lu, Yan Chang, Xindan Li

**Affiliations:** 1 Master training station, General Hospital of Ningxia Medical University, Ningxia, China; 2 Operating room, General Hospital of Ningxia Medical University, Ningxia, China; 3 Department of Gynecology, General Hospital of Ningxia Medical University, Ningxia, China; 4 College of Nursing, Ningxia Medical University, Ningxia, China; 5 Department of Nursing, General Hospital of Ningxia Medical University, Ningxia, China; 6 Department of Hematology, General Hospital of Ningxia Medical University, Ningxia, China; 7 Department of Orthopedics, General Hospital of Ningxia Medical University, Ningxia, China; Shiraz University of Medical Sciences, IRAN, ISLAMIC REPUBLIC OF

## Abstract

**Background:**

Telehealth has become an increasingly important approach in healthcare delivery, particularly in improving accessibility, continuity, and efficiency of nursing services. This study investigated the status and influencing factors of telehealth readiness among nurses in Ningxia, China.

**Methods:**

A multicenter cross-sectional study was conducted from September to December 2024 across 57 public hospitals in Ningxia, utilizing a multistage stratified sampling method. Data were collected through questionnaires that included the Chinese version of the Telehealth Readiness Assessment Tool (TRAT), the Cognitive Telehealth Scale (CTS), the Innovative Behavior Scale (IBS), and the Spiritual Climate Scale (SCS). Statistical analyses included univariate, correlation, and hierarchical multiple linear regression analyses.

**Results:**

A total of 1000 valid questionnaires were collected. The participants were 33.16 ± 7.17 years, and 951 (95.1%) were female. The mean total scores for telehealth readiness, CTS, IBS, and SCS were 62.29 ± 13.61, 67.75 ± 15.82, 34.85 ± 8.59, and 15.96 ± 3.07, respectively. Hierarchical multiple linear regression analysis revealed that education level, professional title, years of work experience, monthly income, perceived adequacy of the nursing workforce for telehealth services, CTS, IBS, and SCS were all significantly and positively associated with telehealth readiness (all *P* < 0.05), explaining 51.2% of the variance in telehealth readiness.

**Conclusion:**

Telehealth readiness among nurses in Ningxia was moderate and influenced by demographic characteristics and psychological factors (cognitive, innovative, and spiritual dimensions). These findings highlight the need for multifaceted interventions that focus on competency building and supportive workplace environments to facilitate telehealth adoption.

## Introduction

Telemedicine refers to the delivery of clinical healthcare services at a distance by physicians or other health professionals, using information and communication technologies to support diagnosis, treatment, prevention, follow-up, and clinical decision-making [1]. It has gained increasing acceptance and adoption among healthcare professionals worldwide [2–5]. Telehealth refers to the use of electronic information and telecommunication technologies to facilitate a wide range of remote health-related activities, including clinical care, patient and professional education, public health functions, and health-system administration [6,7]. Its implementation across hospitals, communities, and households has helped overcome temporal and spatial barriers, reduce healthcare costs, and improve the quality of life of home-based users [8–10]. Nevertheless, effective telehealth deployment requires sufficient human resources, with nurses playing a pivotal role [8,11]. However, many nurses remain accustomed to the traditional care models and demonstrate limited awareness and acceptance of emerging telehealth modalities [12]. Telenursing, a core component of telehealth, is defined as the delivery of nursing care and services at a distance, within the registered nurse’s scope of practice, using information and communication technologies or telecommunication systems to assess, monitor, educate, support, and coordinate care for individuals or populations [13] and aims to enhances the accessibility and quality of healthcare services.

Telehealth readiness refers to the degree to which users, healthcare institutions, and systems are prepared to engage with and implement telehealth applications effectively [14–17]. As the main performers of telehealth services, nurses’ readiness is critical for the success of telehealth initiatives [12,18,19]. Growing evidence indicates that nurse-led telehealth can enhance efficiency and support cost-effective healthcare delivery [18]. Nurses’ telehealth readiness is influenced by various factors, including clinical experience, willingness to adopt new service models, organizational support, and policy environment [20]. In China, nurse-led telehealth refers to a healthcare delivery model centered on nurses that utilizes remote communication technologies (such as video, telephone, or mobile health platforms) to provide remote assessment, management, and monitoring of patients’ health, and remains in its early exploratory phase [21]. Innovative behavior is a key determinant of nurses’ telehealth readiness, promoting adaptability and willingness to integrate telehealth services in their practice [12,22,23]. Furthermore, a positive spiritual climate may strengthen nurses’ occupational coping efficacy, enhance professional fulfillment, foster inner harmony, and boost work motivation [24]. Nonetheless, empirical evidence on the interplay between these psychosocial variables and telehealth readiness remains limited.

The existing literature on nurses’ telehealth readiness, both in China and internationally, shows that most studies are concentrated in developed regions and major metropolitan centers [20,23,25]. Findings suggest that nurses in North America, Europe, Australia, and developing areas of China generally exhibit moderate to high readiness levels, supported by stronger technological infrastructure, structured training, and favorable policy frameworks [20,23]. Despite this progress, persistent barriers include limited technical proficiency, uneven network infrastructure, and the lack of standardized telehealth curricula, particularly in less developed healthcare regions. Nevertheless, in Africa, systematic reviews have highlighted significant hurdles related to digital infrastructure, funding, and workforce capacity that underpin telehealth readiness [26]. Regional analyses indicate the immense potential of telehealth to improve healthcare access across the continent [26,27]. In contrast, the development of telehealth in Latin America is shaped not only by similar universal barriers but also by its distinct policy frameworks and structural challenges within the healthcare system. Research indicates that advancing value-based healthcare in Latin America requires comprehensive policy reforms, infrastructure investment, and multi-stakeholder collaboration among patients,as well as the public, private, and non-profit sectors [28]. In China, most published studies have focused on economically advanced provinces such as Beijing, Shanghai, Guangdong, and Zhejiang, reporting promising pilot outcomes while highlighting pronounced urban-rural disparities in digital literacy and readiness [20,23,25,29,30]. This geographic concentration leaves significant gaps in evidence regarding telehealth readiness in underdeveloped regions [31–33].

Ningxia, situated in western China, faces an uneven distribution of healthcare resources, pronounced urban-rural disparities, and a relatively low per capita allocation of healthcare resources. The region’s aging population and high prevalence of chronic diseases further underscore the need for telehealth development. However, systematic data on nurses’ telehealth readiness in this setting remain lacking. Accordingly, this study aimed to assess the current status of and factors influencing telehealth readiness among nurses in Ningxia, China.

## Methods

### Study design and population

This cross-sectional study was conducted in Ningxia, China, from September 2^nd^ to December 30^th^, 2024. A multistage stratified sampling method was used to survey nurses from secondary and tertiary public hospitals. It was stratified into two levels by hospital grade, referring to 38 secondary and 19 tertiary public hospitals. In the initial stage, approximately one-third of these hospitals were randomly selected, culminating in the inclusion of 6 tertiary and 12 secondary general hospitals. In the subsequent stage, all eligible nurses employed at the selected hospitals were invited to participate. The inclusion criteria were (1) regular employment in the surveyed hospitals with a minimum of one year of nursing experience in a frontline clinical department and (2) possession of a valid Chinese registered nurse license. The study protocol received approval from the Medical Ethics Committee of the General Hospital of Ningxia Medical University Hospital (Approval No. 2023-047-01). Written informed consent was obtained from all participants prior to data collection.

### Data collection

Before data collection, all members of the research team underwent standardized training that encompassed the study objectives, procedures, questionnaire administration, and confidentiality requirements. The researchers engaged with the nursing departments of the selected hospitals to elucidate the study’s purpose and obtain the necessary approvals. Questionnaires were subsequently distributed in hospital wards, accompanied by standardized instructions that delineated the study’s objectives, relevant concepts, and completion requirements. Confidentiality of responses was assured. To maintain data integrity, each mobile phone number or IP address was restricted to a single submission, questionnaires could only be submitted upon completion of all items, and responses with inconsistent demographic information or erroneous entries were excluded. A total of 1,051 questionnaires were distributed, yielding 1,000 valid responses and a response rate of 95.1%.

### Socio-demographic variables

The demographic factors included gender, age, marital status, and monthly income. The socio-professional factors included hospital level, education level, professional title, years of working experience, nursing grades (defined as dividing nurses into different levels according to their professional ability, educational levels, work experience, and professional titles, and giving them corresponding job responsibilities, authorities, and job requirements; currently, China predominantly adopts a five-tier classification system ranging from N0 to N4), department, administrative position, and responses to the following questions: “Has your hospital implemented telehealth services?”, “Have you received training on telehealth service models?”, “Have you ever used a telehealth platform (e.g., video conferencing, health management applications) to provide services to patients?”, “Are you willing to use information technology to provide telehealth services to patients?”, “Do you understand the telehealth policies issued by the country?”, and “In your opinion, does the nursing workforce in your department meet the requirements for implementing telehealth services?”.

### Instruments

#### Telehealth readiness assessment tools (TRAT).

The TRAT was developed by Jennett et al. [34] in 2003 and was translated and revised into Chinese by Liu et al. [35]. It is used to assess nurses’ adaptability to and preparedness for telehealth services. It comprises three dimensions: core readiness (4 items), engagement readiness (7 items), and structural readiness (6 items), totaling 17 items. A 5-point Likert scale is used, with scores from 1 (“strongly disagree”) to 5 (“strongly agree”). The total score ranges from 17 to 85, with scores between 17 and 60 indicating low readiness, 61 and 80 indicating moderate readiness, and 81 and 85 indicating high readiness. The instrument has demonstrated good reliability and validity, with an overall Cronbach’s α coefficient of 0.861.

#### Cognition of telehealth scale (CTS).

The CTS was developed by Chen et al. [36] in 2010 to assess nurses’ cognitive and understanding levels regarding telehealth. It comprises three dimensions: health promotion awareness (6 items), healthcare competence (6 items), and health management services (5 items), totaling 17 items. A 5-point Likert scale is employed, with scores ranging from 1 (“strongly disagree”) to 5 (“strongly agree”). Higher scores indicate a higher level of telehealth cognition. The scale’s internal consistency demonstrated a Cronbach’s α coefficient of 0.886.

#### Innovative behavior scale (IBS).

The IBS was developed by Bao et al. [37] in 2010 to evaluate nurses’ innovative behavior and awareness in clinical practice. It comprises three dimensions: idea generation (3 items), idea promotion (4 items), and idea realization (3 items), totaling 10 items. It employs a self-assessment format using a 5-point Likert scale (1 = “never,” 5 = “very frequently”), in which nurses rate the frequency of their behaviors in practice. Higher scores indicate stronger innovative behaviors. The scale has demonstrated good reliability, with an overall Cronbach’s α coefficient of 0.906 and dimension-specific coefficients ranging from 0.766 to 0.931.

#### Spiritual climate scale (SCS).

The SCS was developed by Cruz et al. [38] in 2017 and subsequently translated and revised into Chinese by Wu et al. [39]. It is designed to evaluate the psychological atmosphere and the level of support within nurses’ work environments. The scale consists of 4 items and employs a 5-point Likert scale, with scores ranging from 1 (“strongly disagree”) to 5 (“strongly agree”). The final score is calculated by subtracting 1 from the average total score and multiplying the result by 25, yielding a score ranging from 0 to 100. Higher scores reflect a more favorable spiritual climate. The scale’s Cronbach’s α coefficient was 0.833.

### Sample size calculation

The sample size was determined in accordance with Kendall’s criterion [40], which recommends including 5–10 participants per variable. In this study, 65 variables were assessed. Given a projected attrition rate of 10% to 20%, the estimated sample size ranged from 715 to 780 participants.

### Statistical analysis

Continuous data were presented as mean and standard deviation (SD), while categorical data were expressed as n (%). The independent-samples t-test was employed for comparisons between two groups, and one-way analysis of variance (ANOVA) was used for comparisons among multiple groups. To investigate the relationships between telehealth readiness and factors such as telehealth cognition, innovative behavior, and spiritual climate, Pearson correlation analysis was conducted. To evaluate the independent contributions of various predictors to telehealth readiness, a hierarchical multiple linear regression analysis was executed, utilizing the telehealth readiness scale score as the dependent variable. Prior to regression analysis, multicollinearity was examined using variance inflation factors (VIFs) and tolerance values; VIF < 10 and tolerance > 0.1 were considered acceptable. Independent variables were selected based on univariate and correlation analyses (*P* < 0.05) and were incorporated into the model stepwise in accordance with the theoretical framework, thereby permitting the assessment of the incremental explanatory power of each block of variables. Data analysis was performed using SPSS version 25.0 (IBM, Armonk, NY, USA). All statistical tests were two-tailed, with *P* < 0.05 signifying statistical significance.

## Results

### Demographic characteristics and telehealth readiness of nurses

Among the 1,000 respondents, 951 (95.1%) were female, with a mean age of 33.16 ± 7.17 years. The analysis indicated statistically significant differences in telehealth readiness across education level, professional title, monthly income, years of working experience, nursing grade, whether the hospital had implemented telehealth services, whether training on telehealth service models had been received, whether telehealth platforms had previously been used to provide patient care, willingness to use information technology to provide telehealth services, understanding of national telehealth policies, and perception of whether the nursing workforce in the department was sufficient to implement telehealth services (all *P* < 0.05) (Table 1).

**Table 1:** Demographic characteristics and univariate analysis of Telehealth readiness among nurses (N = 1,000).

Variables	n (%)	Telehealth Readiness Scale, mean ± SD	*P*
Hospital Level			0.904
Tertiary	578 (57.8)	62.34 ± 12.85	
Secondary	422 (42.2)	62.23 ± 14.59	
Gender			0.714
Male	49 (4.9)	70.12 ± 15.81	
Female	951 (95.1)	67.63 ± 15.82	
Age (years)			0.928
20-29.9	421 (42.1)	62.24 ± 13.49	
30-39.9	431 (43.1)	62.19 ± 14.27	
40-49.9	124 (12.4)	63.05 ± 12.19	
≥ 50	24 (2.4)	61.67 ± 10.77	
Education level			<0.001
Master’s degree or above	16 (1.6)	71.38 ± 12.81	
Bachelor’s degree	776 (77.6)	63.42 ± 12.55	
Junior college	192 (19.2)	58.45 ± 12.55	
Secondary technical school or below	16 (1.6)	44.63 ± 16.46	
Professional title			<0.001
Chief nurse	13 (1.3)	67.39 ± 13.72	
Deputy chief nurse	87 (8.7)	73.77 ± 13.04	
Nurse-in-charge	309 (30.9)	63.05 ± 14.22	
Nurse practitioner	433 (43.3)	60.05 ± 12.06	
Staff nurse	158 (15.8)	60.24 ± 13.41	
Years of working experience			<0.001
0-3	197 (19.7)	59.25 ± 11.58	
4-6	93 (9.3)	60.13 ± 13.93	
7-10	253 (25.3)	60.34 ± 12.04	
≥ 11	457 (45.7)	65.13 ± 14.62	
Marital status			0.879
Unmarried	230 (23)	62.40 ± 13.47	
Married	748 (74.8)	62.30 ± 13.50	
Divorced or other	22 (2.2)	60.86 ± 18.58	
Monthly income (CNY)			<0.001
2000-3999	212 (21.2)	59.57 ± 13.75	
4000-5999	442 (44.2)	60.10 ± 12.75	
6000-7999	215 (21.5)	67.11 ± 14.16	
≥ 8000	130 (13)	66.05 ± 12.27	
Nursing grade			0.015
N4	76 (7.6)	67.09 ± 13.39	
N3	258 (25.8)	61.97 ± 13.10	
N2	335 (33.5)	61.13 ± 13.40	
N1	197 (19.7)	62.71 ± 14.67	
N0	134 (13.4)	62.52 ± 13.15	
Department			0.765
Obstetrics and Gynecology	56 (5.6)	62.30 ± 12.90	
Pediatrics	44 (4.4)	64.57 ± 15.58	
Emergency Department	44 (4.4)	62.02 ± 13.66	
Intensive Care Unit	75 (7.5)	61.80 ± 12.86	
Operating Room	95 (9.5)	63.21 ± 15.82	
Other	198 (19.8)	63.22 ± 13.66	
Administrative position			0.600
Yes	68 (6.8)	64.09 ± 13.24	
No	932 (93.2)	62.16 ± 13.63	
Has your hospital implemented telehealth services?			<0.001
Yes	281 (28.1)	64.80 ± 15.49	
No	719 (71.9)	61.31 ± 12.68	
Have you received training on telehealth service models?			<0.001
Yes	298 (29.8)	65.74 ± 15.28	
No	702 (70.2)	60.83 ± 12.56	
Have you ever used a telehealth platform (e.g., video conferencing, health management applications) to provide services to patients?			<0.001
Yes	367 (36.7)	64.95 ± 14.99	
No	633 (63.3)	60.75 ± 12.49	
Are you willing to use information technology to provide telehealth services to patients?			<0.001
Yes	794 (79.4)	63.42 ± 13.34	
No	206 (20.6)	57.97 ± 13.80	
Do you understand the telehealth policies issued by the country?			<0.001
Yes	548 (54.8)	64.66 ± 14.48	
No	452 (45.2)	59.42 ± 11.86	
In your opinion, does the nursing workforce in your department meet the requirements for implementing telehealth services?			<0.001
Yes	477 (47.7)	64.31 ± 14.17	
No	523 (52.3)	59.54 ± 12.46	

### Telehealth readiness of nurses

The total telehealth readiness score of nurses in Ningxia was 62.29 ± 13.61 points. From lowest to highest, the scores of each dimension were core readiness (13.51 ± 3.70), structural readiness (22.20 ± 5.30), and engagement readiness (26.58 ± 5.84) (Table 2).

**Table 2:** Dimension scores of telehealth readiness among nurses (n = 1000).

Variables	Total score (mean ± SD)	Item average score (mean ± SD)
Readiness for Telehealth	62.29 ± 13.61	3.72 ± 0.76
Core readiness	13.51 ± 3.70	3.38 ± 0.92
Structural readiness	22.20 ± 5.30	3.72 ± 0.76
Engagement readiness	26.58 ± 5.84	3.80 ± 0.83

### Correlation between telehealth readiness and the scores of CTS, IBS, and SCS among nurses

The total scores of CTS, IBS, and SCS among nurses in Ningxia were 67.75 ± 15.82, 34.85 ± 8.59, and 15.96 ± 3.07, respectively (Table 3). Pearson correlation analysis revealed that telehealth readiness was positively correlated with the scores of CTS (r = 0.587), IBS (r = 0.508), and SCS (r = 0.547), with all correlations being statistically significant (all *P* < 0.01) (Table 4).

**Table 3:** The scores of CTS, IBS, and SCS among nurses (N = 1,000).

Variables	Total score (mean ± SD)	Item average score (mean ± SD)
CTS	67.75 ± 15.82	3.98 ± 0.93
Health Promotion Awareness	23.99 ± 5.69	4.00 ± 0.95
Health Care Competence	23.75 ± 5.69	3.96 ± 0.95
Health Management Services	20.02 ± 4.79	4.00 ± 0.96
IBS	34.85 ± 8.59	3.49 ± 0.86
Idea Generation	10.48 ± 2.60	3.49 ± 0.86
Idea promotion	13.90 ± 3.58	3.48 ± 0.89
Idea Implementation	10.47 ± 2.76	3.49 ± 0.92
SCS	15.96 ± 3.07	3.99 ± 0.77

Abbreviations: CTS, the Cognition of Telehealth Scale; IBS, the Innovative Behavior Scale; SCS, the Spiritual Climate Scale.

**Table 4:** Correlations of telehealth readiness with the total scores of CTS, IBS, and SCS.

Variables	Telehealth Readiness	CTS	IBS	SCS
Telehealth Readiness	1.000			
CTS	0.587**	1.000		
IBS	0.508**	0.421**	1.000	
SCS	0.547**	0.492**	0.503**	1.000

Abbreviations: CTS, the Cognition of Telehealth Scale; IBS, the Innovative Behavior Scale; SCS, the Spiritual Climate Scale. **P < 0.01.

### Multiple regression analysis of factors influencing telehealth readiness among nurses

Before stepwise multiple linear regression analysis, multicollinearity diagnostics were conducted. The results showed that the VIF values for the independent variables ranged from 1.039 to 2.439, all below the threshold of 10, and the tolerance values ranged from 0.410 to 0.962, all above the threshold of 0.2. These findings indicate that there was no serious multicollinearity among the independent variables. The categorical variables were processed as dummy variables (Table 5).

**Table 5:** Coding scheme for independent variables.

Variables	Coding method
Education level	Secondary technical school = 1; Junior college = 2; Bachelor’s degree = 3; Master’s degree or above = 4
Professional title	Staff nurse = 1; Nurse practitioner = 2; Nurse-in-charge = 3; Deputy chief nurse = 4; Chief nurse = 5
Monthly income (CNY)	2,000–3,999 = 1; 4,000–5,999 = 2; 6,000–7,999 = 3; > 8,000 = 4
Years of working experience	0–3 = 1; 4–6 = 2; 7–10 = 3; ≥ 11 = 4
Nursing grade	N0 = 1; N1 = 2; N2 = 3; N3 = 4; N4 = 5
Whether the hospital has implemented telehealth services	Yes = 1; No = 0
Whether training on the telehealth service model has been received	Yes = 1; No = 0
Whether telehealth platforms have ever been used to provide services to patients	Yes = 1; No = 0
Willingness to use information technology to provide telehealth services to patients	Yes = 1; No = 0
Understanding of telehealth policies issued by the state	Yes = 1; No = 0
Perception of whether the nursing workforce in the department meets the requirements for implementing telehealth services	Yes = 1; No = 0
CTS	Entered as original value
IBS	Entered as original value
SCS	Entered as original value

Abbreviations: CTS, the Cognition of Telehealth Scale; IBS, the Innovative Behavior Scale; SCS, the Spiritual Climate Scale.

Hierarchical multiple linear regression was performed with the telehealth readiness score as the dependent variable. In the first layer (Model 1), the variables with statistically significant differences in the univariate analysis were included (education level, professional title, nursing grade, years of working experience, monthly income, etc.). In the second layer (Model 2), psychological variables were also included (CTS, IBS, and SCS scores). The results showed that the socio-demographic variables explained 19.2% of the variance (ΔR^2^ = 0.192, *P* < 0.001). After adding psychological variables, the explanatory power increased to 51.2% (ΔR^2^ = 0.320, *P* < 0.001). Education level, professional title, years of working experience, monthly income, and perception of whether the nursing workforce in the department was sufficient to implement telehealth services were all significantly and positively associated with telehealth readiness. Additionally, the scores of CTS, IBS, and SCS were also significantly and positively associated with telehealth readiness. In contrast, nursing grade was significantly and negatively associated with telehealth readiness (β = −0.276, *P* < 0.001) (Table 6).

**Table 6:** Hierarchical multiple linear regression analysis of factors associated with telehealth readiness among nurses.

Independent variables	Model 1			Model 2		
	B	β	t	B	β	t
Education level	5.160	0.181	6.255***	3.273	0.115	5.057***
Professional title	3.685	0.242	6.111***	1.986	0.131	4.174***
Years of working experience	2.351	0.200	4.912***	1.534	0.130	4.102***
Monthly income	1.124	0.111	3.792***	0.464	0.046	1.998***
Nursing grade	−3.304	−0.276	−6.316***	−1.818	−0.152	−4.399***
Has your hospital implemented telehealth services?	−0.363	−0.012	−0.334	−0.134	−0.004	−0.158
Have you received training on telehealth service models?	−1.597	−0.054	−1.387	−1.441	−0.048	−1.609
Have you ever used a telehealth platform to provide services to patients?	−0.729	−0.026	−0.728	−.017	−0.001	−0.022
Are you willing to use information technology to provide telehealth services to patients?	−1.581	−0.047	−1.464	1.378	0.041	1.624
Do you understand the telehealth policies issued by the country?	−1.828	−0.067	−1.957	−0.756	−0.028	−1.038
In your opinion, does the nursing workforce in your department meet the requirements for implementing telehealth services?	−1.292	−0.021	−0.062	−1.406	−0.052	−1.998*
CTS	—	—	—	0.302	0.351	13.301***
IBS	—	—	—	0.289	0.183	6.728***
SCS	—	—	—	0.975	0.220	7.879***
R^2^	0.201			0.519		
ΔR^2^	0.192			0.512		
F	22.534			75.764		

Abbreviations: *P < 0.05; ***P < 0.001; B, unstandardized coefficient; β, beta standardized coefficient; t, beta t-test statistic; F, variance of the regression equation; R^2^, cumulative variance explained; ΔR^2^, variance increment explained. CTS, the Cognition of Telehealth Scale; SCS, the Spiritual Climate Scale; IBS, the Innovative Behavior Scale.

## Discussion

This multicenter cross-sectional study examined telehealth readiness and its influencing factors among 1000 nurses in Ningxia, revealing a moderate overall level of readiness. Several demographic characteristics (education level, professional title, years of experience, monthly income, and perceived adequacy of the nursing workforce) and psychological factors (cognitive, innovative, and spiritual dimensions) were associated with telehealth readiness, while nursing grades showed a negative correlation. In addition, psychological factors substantially increased the model’s explanatory power.

In this study, the level of telehealth readiness was higher than reported for community health service center nurses and tertiary-hospital nurses in prior Chinese studies [14,19], which may be linked to more favorable human resource allocation and policy support [20]. Indeed, recent policy initiatives around “Internet-based Healthcare” have promoted nurse training via remote and online formats, clarified telehealth service models and risk oversight, and raised public awareness, which likely contributed to the comparatively higher readiness observed among nurses in Ningxia [33,41,42]. The core readiness dimension had the lowest average item score, suggesting that nurses may lack practical experience with telehealth models. Consistent with earlier findings, two major barriers to nurses’ telehealth adoption were insufficient professional training and difficulties operating equipment [43]. Governments and healthcare institutions should develop structured curricula and infrastructure that incorporate telehealth demonstrations, operational training, and simulation-based practice to strengthen nurses’ preparedness, confidence, and competence [44].

Higher telehealth cognition improves understanding of relevant technologies, services, and workflows, facilitating effective use and support of telehealth, which, in China, aligns with community-based older adult care and offers a novel model for disease management and nurse–patient relationship building [45]. Innovative behavior, encompassing both self-efficacy and observed creativity in practice, plays a significant role in shaping nursing practice [20,23,32]. Nurses who score higher on the Innovative Behavior Scale tend to embrace new methods and information technologies more readily, actively seek out professional development resources, and explore novel solutions to clinical challenges [23,46]. A favorable spiritual climate was also positively associated with telehealth readiness. Such a climate promotes emotional expression and humanistic care, mitigates burnout, and improves team collaboration [47]. When nurses feel respected, empowered, and aligned with organizational values, their telehealth readiness and professional engagement rise, a pattern supported by Chinese and international research [13,20,23,32].

Nurses’ professional competence (educational background, professional title, and years of experience) and career compensation (income) also influence their readiness for telehealth care, which aligns with previous research [12,20,48]. Nurses with higher educational attainment, senior titles, and longer professional tenure have generally received more systematic nursing education and possess a deeper knowledge base, stronger academic and clinical skills, all of which enable them to better understand and apply new technologies [48,49]. Nursing grade, a core component of hospital nursing management, was negatively correlated with telehealth readiness [49,50]. In this study, nurses at levels N0 and N1 scored higher than those at N2 and N3 but lower than N4, suggesting that early-career nurses may have stronger enthusiasm and learning motivation for medical apps, while N4 nurses, as recognized experts, actively acquire new knowledge and respond to rapid advances in information technology [20,51,52].

The Ningxia context is crucial to interpreting these findings. Ningxia has relatively scarce medical resources, with low per capital availability of healthcare personnel, facilities, and beds, and lags behind the national average in resource allocation, while eastern provinces have denser and faster-developing systems [53]. The region also shows pronounced urban-rural disparities in access, affordability, and infrastructure, alongside population aging and high chronic disease burden, which intensify pressure on the system and highlight telehealth as a scalable solution [53,54]. Thus, telehealth policies for western China should be tailored to local realities.

Several limitations should be noted. First, sampling from selected secondary and tertiary general hospitals may restrict generalizability. Second, cross-sectional design permits assessment of associations between demographic/individual factors and readiness, but not temporal change or causality. Third, focusing on a single Chinese region limits external validity, and self-administered questionnaires may introduce recall and social desirability bias. Fourth, nursing is a female-dominated profession globally, but the proportion of women in this sample (95.1%) was higher than typical workforce estimates, which show that women account for about 77%−90% of nurses, depending on region and setting [53]. This overrepresentation of female nurses means the study’s results primarily reflect the experiences, attitudes, and behaviors of women and may not adequately capture perspectives more common among male nurses. Male nurses may encounter unique challenges, perspectives, or facilitators in telehealth adoption [55,56]. Future studies can examine telehealth readiness across more diverse gender groups in healthcare and use longitudinal and mixed-methods designs to examine barriers and mechanisms shaping nurses’ telehealth implementation.

## Conclusion

Nurses in Ningxia exhibited moderate telehealth readiness influenced by education level, professional title, nursing grade, years of experience, income, telehealth cognition, innovative behavior, and spiritual climate. The findings underscore that enhancing telehealth readiness extends beyond technical training and requires a holistic strategy. Strengthening the professional competence of telehealth nursing teams in light of local resource constraints will require comprehensive, practice-oriented continuing education and a focus on competency development and supportive workplace environments.
